# Contribution of green organs to grain weight in dryland wheat from the 1940s to the 2010s in Shaanxi Province, China

**DOI:** 10.1038/s41598-021-82718-y

**Published:** 2021-02-09

**Authors:** Yingying Sun, Suiqi Zhang, Jiakun Yan

**Affiliations:** 1 Key Laboratory of Degraded and Unused Land Consolidation Engineering the Ministry of Natural Resources of China, Shaanxi Provincial Land Engineering Construction Group Co., Ltd., Xi’an, 710075 China; 2 State Key Laboratory of Soil Erosion and Dryland Farming On the Loess Plateau, Northwest A&F University, 26# Xinong Road, Yangling, 712100 China; 3 College of Life Science, Yulin University, Yulin, 719000 China

**Keywords:** Plant evolution, Plant physiology

## Abstract

Eight dryland winter wheat cultivars (*Triticum aestivum* L.), which were widely cultivated from the 1940s to the 2010s in Shaanxi Province, China, were selected and grown in plots, and two water treatments (irrigation and drought) were used to identify the contribution of ears, leaves and stems to grain weight and grain number associated with cultivar replacement. The plant height and stem dry weight of the dryland wheat decreased significantly during the cultivar replacement process, but there was a remarkable increase in the dry matter translocation of stems under irrigation. Shaded-ear and defoliation treatment could decrease the grain number and grain weight, and the grain weight was more influenced. Both the leaf and ear are important photosynthetic sources for dryland wheat, and the contribution of ear assimilates showed a significant increase over time; however, the contribution of leaf assimilates showed a negative correlation with cultivation over time. The accumulation of stem assimilates and ear photosynthesis both increased the grain weight potential. In the future breeding process, cultivars with more assimilates stored in the stem and greater assimilative capacity of ears, especially a greater contribution of ear assimilates, are expected to increase the grain yield.

## Introduction

There are three sources for the accumulation of dry matter in wheat grains: photosynthetic products after anthesis and temporary substances of vegetative organs before and after anthesis^1^. Although the ratios of the three resources differ with cultivars and planting conditions, it is generally accepted that photosynthetic products after anthesis play an important role in grain weight accumulation. Most studies assessing the photosynthetic products after anthesis have focused on leaf photosynthesis, especially flag leaf photosynthesis^2–5^, and few studies have focused on the stem and ear. Evans (1992)^6^ confirmed that ear assimilates contribute little to the grain weight, although active grain respiration was found during grain-filling and the ear could refix the CO_2_ released by respiration^7^; thus, the apparent photosynthetic rate may be significantly lower than its actual contribution. The contribution of ear assimilates to grain weight after anthesis is estimated to be between 10 and 76%^1,8,9^. Sanchez-Bragado et al. (2014)^10^ confirmed even higher ear contributions, ranging from 91 to 100% and from 49 to 82% for old landraces and modern cultivars, respectively.

The contribution of the ear to grain weight depends on the environmental conditions and genotypes^10^. Photosynthesis was much higher in the ear than in the flag leaf under well-watered conditions, and as water stress developed, photosynthesis decreased less in the ear than in the flag leaf^11^. Moderate water deficits may enhance the role of the ear as a photosynthetic source^12^, which could result in a higher contribution by the ear than by the flag leaf^13,14^. It has been confirmed that there are differences among genotypes in terms of the contribution of the ear to grain weight^15^. Cultivars with awns, i.e., threadlike extensions of the lemma, have up to 50% increased surface areas of the ear in bread wheat^13^ and up to 60% increased surface areas of the ear in longer-awned, durum wheat^16^, which may result in an increased contribution of the ear^15,17^. Genotypes with higher drought resistance could maintain high ear photosynthetic capacity^18^.

Preanthesis stem assimilates are also an important “source” for grain-filling^19,20^. When there are adverse stresses, such as drought, the normal photosynthesis process of wheat is disrupted and the photosynthetic products cannot meet the needs of canopy respiration and grain-filling; thus, temporary assimilates in the organs are particularly important^21,22^. Richards (1992) confirmed that there was no significant difference between nondwarf and dwarf cultivars in terms of the dry matter storage of stems or the dry matter translocation efficiency of stems^23^. The results of Maydup et al. (2012) indicated that the contribution of stem assimilates of dwarf and semidwarf cultivars decreased significantly relative to that of nondwarf cultivars^15^. Although dwarf genes result in decreased temporary assimilates in single stems, the dry matter translocation efficiency of stems increases and contributes more to grain weight^24,25^.

Although an increasing number of studies have focused on nonleaf organ photosynthetic characteristics and how they influence grain yield, only a few studies have investigated how the ear, stem and leaf influence the grain weight during cultivar replacement, and fewer studies have assessed how environmental conditions, such as water deficit, influence the contribution of photosynthetic organs. Shaanxi Province is located in an arid and semiarid region of China, and it has been confirmed that there is no significant difference in leaf photosynthetic characteristics after anthesis between modern and older cultivars planted here. Additionally, there is a poor correlation with yield increase^26^. Thus, we hypothesize that the contributions of ears and stems to grain weight all increase with cultivar replacement in Shaanxi Province. Cultivars cultivated from the 1940s to the 2010s were selected and defoliation, and shaded ear treatments were designed. The contributions of stem, ear and leaf assimilates to grain weight and grain number after anthesis were compared to identify how different green organs influence grain weight accumulation and to help select high-yield wheat cultivars.

## Materials and methods

### Plant material and experimental design

Eight cultivars planted on a large scale in Shaanxi Province were selected (Table 1). Field experiments were conducted in Yangling, Shaanxi Province, in northwestern China (34° 16′ 56.24″ N, 108° 4′ 27.95″ E; 460 m above sea level) over the winter-spring growing season (i.e., October–June of the following year between 2012 and 2014). Seeds were planted on 12 October 2012 and 10 October 2013 and harvested in late May to early June of the next year (Table 2).

**Table 1:** Recorded traits of selected dryland winter wheat in Shaanxi Province during the 1940s–2010s.

Cultivar	Planting decade in Shaanxi	Pedigree	Dwarf genes	Breeding site	Grain yield (kg ha^−1^)	Drought tolerance
Mazha	1940s	Landrace	None	Shaanxi Province	3526	Strong
Bima1	1950s	Mazha/quality	None	Shaanxi Province	4089	Strong
Fengchan3	1960s	Danmai1/Xinong 6028 × Bima1	None	Shaanxi Province	4954	Strong
Taishan1	1970s	54,405(Bima4 × Zaoshu1)/Orofen	*RhtD1b*	Shandong Province	4979	Medium
Xiaoyan6	1980s	(ST2422 × 464)/Xiaoyan96	*Rht-B1b* + *Rht8*	Shaanxi Province	5247	Strong
Jinmai33	1990s	Pingyang79391(Naixue × 5017)036 × 76–1256)/Pingyang76262	None	Shanxi Province	4265	Strong
Changwu134	2000s	(Changwu131 × Xiaohei96)F1/Changwu131)F4/(Jinghua3/NS2761)F1	*Rht-B1b*	Shaanxi Province	5164	Strong
Changhan58	2010s	Changwu112/PH82-2	*Rht-B1b*	Shaanxi Province	5281	Strong

**Table 2:** Anthesis date and harvest date of all cultivars in the experimental seasons.

Experimental season	Water treatments	Mazha	Bima1	Fengchan3	Taishan1	Xiaoyan6	Jinmai33	Changwu134	Changhan58
**2012–2013**									
Anthesis date	Irrigation	29 April	29 April	30 April	30 April	28 April	29 April	29 April	29 April
	Drought	27 April	27 April	27 April	26 April	26 April	26 April	26 April	27 April
Harvest date	Irrigation	4 June	4 June	3 June	3 June	4 June	3 June	4 June	5 June
	Drought	29 May	28 May	26 May	27 May	26 May	28 May	26 May	29 May
**2013–2014**									
Anthesis date	Irrigation	2 May	30 April	1 May	1 May	29 April	30 April	1 May	1 May
	Drought	28 April	28 April	26 April	27 April	24 April	24 April	24 April	28 April
Harvest date	Irrigation	13 June	12 June	12 June	12 June	12 June	11 June	13 June	13 June
	Drought	8 June	6 June	5 June	5 June	5 June	5 June	6 June	6 June

The soil consists of Earth-cumuli-Orthic Anthrosols with a deep profile and is considered suitable for crop production. During the fallow period of each year, *Vigna radiata* (Linn.) Wilczek were planted and irrigation was provided to regulate the soil water and fertilization. Before the two experimental seasons, the average soil nutrient contents at a depth of 0–20 cm were as follows: organic matter, 18.95 g kg^−1^; total nitrogen, 1.02 g kg^−1^; alkali-hydrolyzable nitrogen, 72.03 mg kg^−1^; total phosphorus, 0.92 g kg^−1^; rapidly available phosphorus, 18.95 g kg^−1^; and available potassium, 152.46 g kg^−1^. Urea (N 150 kg ha^−1^) and calcium superphosphate (P_2_O_5_, 120 kg ha^−1^) were provided prior to planting, and no topdressing was applied before harvest.

**Figure 1:**
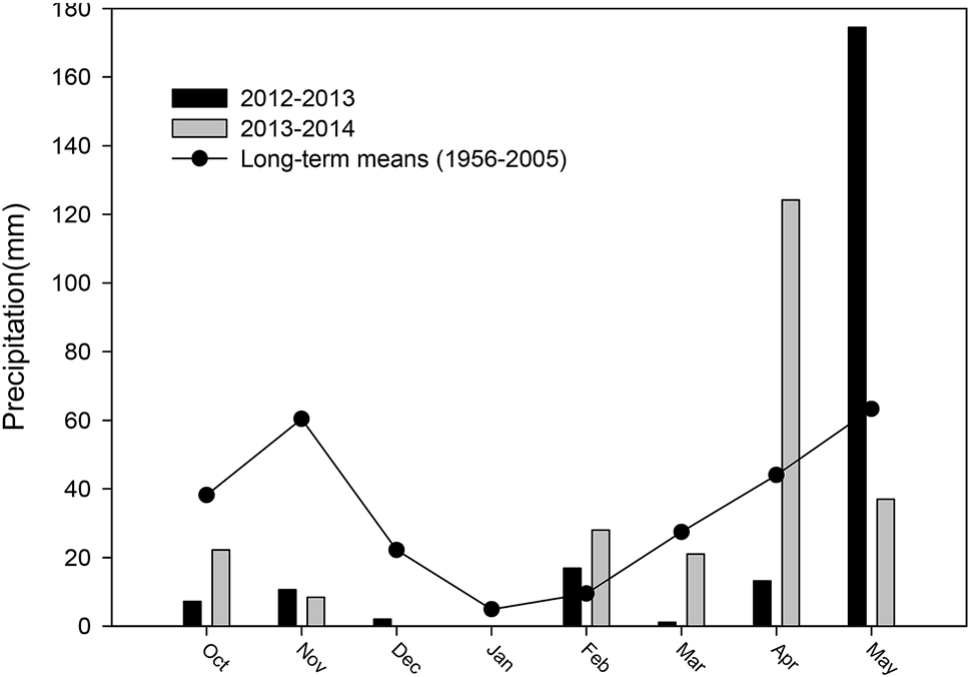
Precipitation during the experimental period (October–May of the following year) between 2012 and 2014 compared with the long-term means (1956–2005) at the experimental site.

Two water treatments (irrigation, Ir; drought, D) were implemented (Table 3). To ensure the growth of wheat at the seedling stage, normal rainfall without irrigation was provided to the wheat under drought treatment before the jointing stage. The drought treatment was administered under a rainout shelter to prevent the effects of precipitation after the jointing stage. A rain gauge with a diameter of 10 cm and height of 60 cm was used to record the precipitation during each growing season (Fig. 1). The volume of all collected water in the rain gauge after each rainfall or snowfall was measured (ml) and converted into precipitation (mm). Cultivars were planted manually in plots (2.2 × 3.3 m per plot; 11 rows that were 20 cm apart; plant spacing of 2 cm). Plots were arranged in randomized blocks with three replicates. Fungicides and pesticides were applied at shooting and grain-filling to prevent attacks by diseases and pests to eliminate the effects of differences in pest resistance between different cultivars. The overhigh plants of Mazha, Bima1 and Jinmai 33 easily led to lodge, so bamboo poles (50–200 cm long) were used to avoid cultivar differences caused by lodging, enabling the ultimate yield potential to be reached.

**Table 3:** Total precipitation during the growth period of 2012–2014 (mm) and the irrigation supplement in the irrigation treatment (mm).

Experimental season	Drought treatment		Irrigation treatment	
	Precipitation (mm)	Precipitation (mm)	Irrigation at tillering stage (mm)	Irrigation at jointing stage (mm)
2012–2013	36.8	225.6	70.0	70.0
2013–2014	22.2	240.7	60.0	0

### Defoliation and ear-shading

Combinations of defoliation and ear-shading were imposed at anthesis in both seasons. The anthesis date of each cultivar was evaluated based on 50% of plants per plot at anthesis. Forty main culms with similar heights and ear sizes among the 50% were selected and labeled in each plot, and they were treated 3 days after anthesis for each cultivar as described below.

- *Control check (CK)* labeled only, without any action (nonshaded ear or defoliation), 10 culms per plot, 30 culms for each cultivar under the same water treatment.
- *Shaded ear* ear was shaded using aluminum foil (several 1-mm holes were arranged in the aluminum foil to ensure gas exchange), 10 culms per plot, 30 culms for each cultivar under the same water treatment.
- *Defoliated* all leaves of the culms were cut from the base, and the leaf sheath was retained, 10 culms per plot and 30 culms for each cultivar under the same water treatment.
- *Shaded-ear + defoliated* all leaves were cut and the ear was shaded, 10 culms per plot and 30 culms for each cultivar under the same water treatment. The number of kernels and the grain weight per ear were measured at maturity.

The ear and leaf contribution (%) to grain weight per spike (GW) was calculated as follows^15,27^:

$$ {\text{Contribution of ear assimilates to grain yield per spike }}\left( {{\text{CEA}}} \right) = \left[ {\left( {{\text{GW of nonshaded ear}} - {\text{GW of shaded ear}}} \right) \, /{\text{ GW of nonshaded ear}}} \right] \, \times { 1}00 $$

$$ {\text{Contribution of leaf assimilates to grain yield per spike }}\left( {{\text{CLA}}} \right) = \left[ {\left( {{\text{GW of nondefoliated}} - {\text{GW of defoliated}}} \right) \, /{\text{ GW of nondefoliated}}} \right] \, \times { 1}00 $$

Ten culms with similar growing conditions were selected, collected and dried to measure the height and dry matter of stems (culms without ears or leaves) at anthesis in each plot; of these samples, all leaves and ears were cut and the leaf sheaths were retained. Plant height was measured from the bottom of a single stem to the tip of the spike. Because stem dry matter may increase continuously after anthesis, stems were collected every 5 days after anthesis until the maximum weight was achieved, and the results were identified as the DM_max_. The height and single-stem weight of four cultivars without dwarf genes (i.e., 1940s, 1950s, 1960s, 1990s) continued to increase after anthesis, reaching the maximum value 15 or 20 days after anthesis. Ten stems were selected from the CK groups to determine the dry matter of stems at maturity (DM_maturity_).

The dry matter translocation of stems (DMT), the dry matter translocation efficiency of stems (DMT_e_, %) and the contribution of stem assimilates (CSACSA, %) were calculated by DM_max_, DM_maturity_ (dry weight of culms without ears or leaves at maturity) and GW (grain weight per spike) of CK as follows^15,27^:

$$ \begin{aligned} {\text{DMT }} & = {\text{ DM}}_{{{\text{max}}}} - {\text{ DM}}_{{{\text{maturity}}}} \\ {\text{DMT}}_{{\text{e}}} & = \left( {{\text{DMT }}/{\text{ DM}}_{{{\text{max}}}} } \right) \, \times { 1}00 \\ {\text{CSA }} & = \left( {{\text{DMT }}{/}{\text{ GW of CK}}} \right) \, \times { 1}00 \\ \end{aligned} $$

### Statistical analysis

The interaction of different factors (cultivars, defoliation and ear-shading, water treatment, and experimental seasons) with grain number and grain weight per ear was analyzed using a general linear model. To avoid differences in climatic conditions between the two years in the experiment, the data for each year are plotted separately. An average value was calculated by all 30 samples in three plots for each cultivar under same water treatment (N = 30). Grain number and grain weight per ear differences between groups were examined using one-way analysis of variance (ANOVA). When ANOVA results showed significant differences, the differences between groups were detected by Fisher's LSD postmortem. Correlations between phenotypic traits and yield elements, as well as between the contribution of different organs and planting decades, were all determined using Pearson’s test. SPSS 19.0 software ((IBM Corp: Armonk, NY, USA).) was used to perform the analyses.

## Results

### Plant height and single-stem weight of dryland winter wheat cultivars from different decades

**Figure 2:**
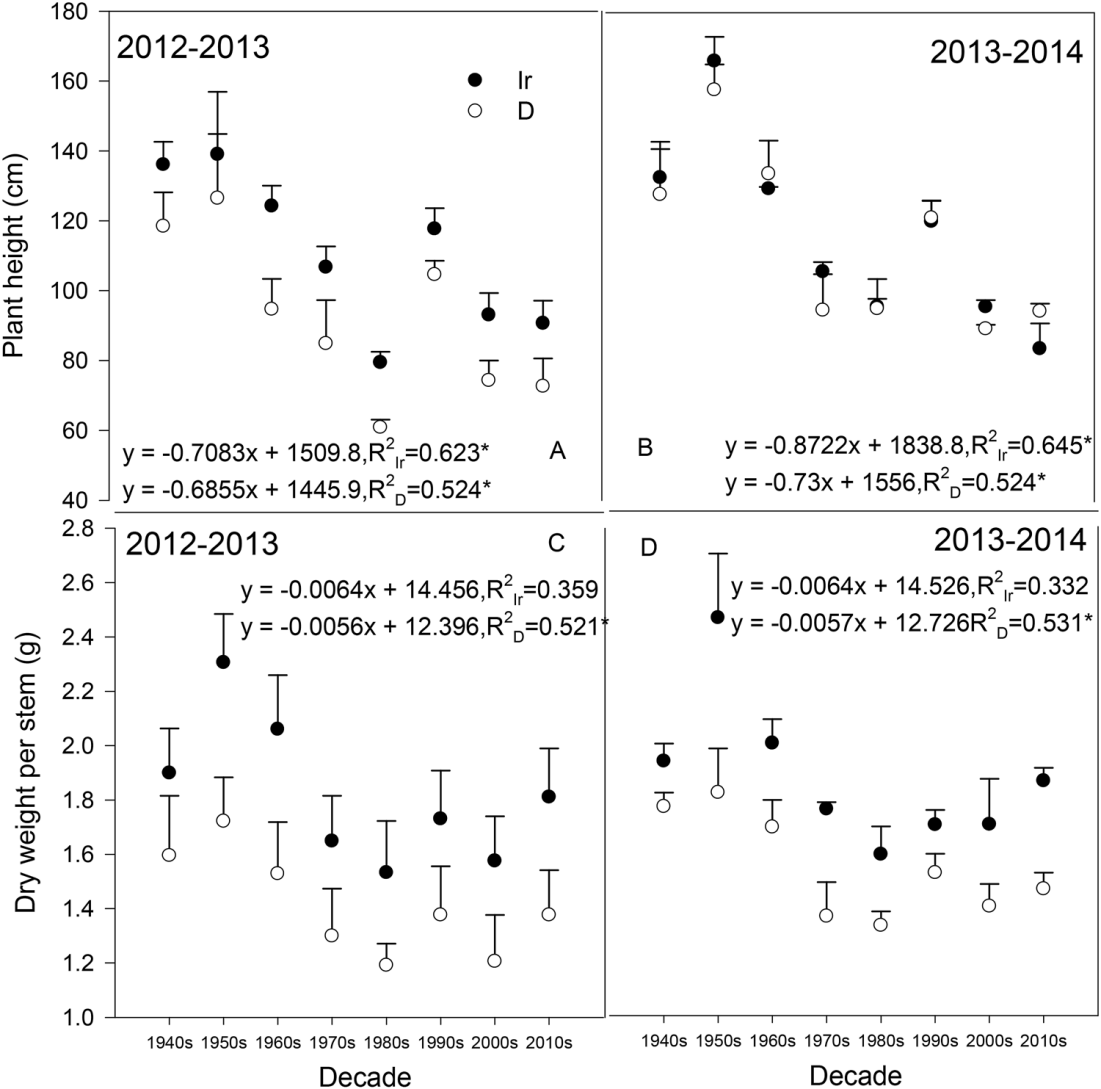
Plant height and stem dry weight at anthesis in 2012–2013 and 2013–2014. The data represent the mean ± SE and sample number (N) = 30; * significant at *P* < 0.05; Ir indicates the irrigation treatment; D indicates the drought treatment.

In both planting seasons, the plant height tended to be generally shorter and the stem dry weight decreased with the cultivar replacement (Fig. 2), but the stem dry weight downtrend was not significant in irrigation treatment (P < 0.05). All cultivars showed significantly higher height and stem dry weight under irrigation than in the drought treatment in the 2012–2013 season (Fig. 2A,C), and the difference between the different water treatments was smaller in the 2013–2014 season (Fig. 2B,D), which may be due to a smaller moisture difference in the water treatments resulting from the abundance of precipitation during the crop rotation of *Vigna radiata* (Linn.) Wilczek.

### Effect of shaded-ear and defoliation treatments on grain number and grain weight per ear

According to the interaction analysis of different factors on grain number and grain weight per spike (Table 4), almost all the factors, including cultivars, defoliation and ear-shading, water treatment, and experimental seasons, can interact with each other to a significant level at *P* < 0.01. Therefore, we performed variance analysis of different shaded-ear and defoliation treatments of the same cultivar under the same experimental season and water treatment.

**Table 4:** Contribution of different factors to grain number and grain weight per spike.

Items	Grain number per spike					Grain weight per spike				
	Type IIISS	df	Mean square	F	Sig	Type IIISS	df	Mean square	F	Sig
Experimental season	2410.0	1	2410.0	71.9	0.000**	31.7	1	31.7	454.5	0.000**
Water	11,047.8	1	11,047.8	329.8	0.000**	19.9	1	19.9	285.5	0.000**
Cultivar	62,797.7	10	8971.1	267.8	0.000**	49.9	10	7.1	102.0	0.000**
Defoliation and ear-shading	3052.7	3	1017.6	30.4	0.000**	105.1	3	35.0	501.6	0.000**
Experimental season × water	4850.7	1	4850.7	144.8	0.000**	8.3	1	8.3	119.3	0.000**
Experimental season × cultivar	15,635.6	10	2233.7	66.7	0.000**	16.5	10	2.4	33.7	0.000**
Experimental season × defoliation and ear-shading	499.1	3	166.4	5.0	0.002**	2.8	3	0.9	13.2	0.000**
Water × cultivar	1900.6	10	271.5	8.1	0.000**	2.3	7	0.3	4.6	0.000**
Water × defoliation and ear-shading	95.3	3	31.8	1.0	0.416	2.4	3	0.8	11.6	0.000**
Cultivar × defoliation and ear-shading	2278.0	30	108.6	3.2	0.000**	26.6	30	1.3	18.1	0.000**
Experimental season × water × cultivar	3567.2	10	509.6	15.2	0.000**	3.2	10	0.5	6.5	0.000**
Experimental season × water × defoliation and ear-shading	1348.4	3	449.5	13.4	0.000**	3.0	3	1.0	14.2	0.000**
Experimental season × cultivar × defoliation and ear-shading	1849.6	30	88.1	2.6	0.000**	12.5	30	0.6	8.5	0.000**
Water × cultivar × defoliation and ear-shading	1622.4	30	77.3	2.3	0.001**	2.4	30	0.1	1.6	0.037*
Experimental Season × water × cultivar × defoliation and ear-shading	1753.9	30	83.5	2.5	0.000**	3.7	30	0.2	2.5	0.000**
Error	73,529.3		33.5			153.2		0.1		
Aggregate	3,001,875.0					3638.2				
Corrected aggregate	181,901.7					434.7				

*Significant at *P* < 0.05; **significant at *P* < 0.01.

**Figure 3:**
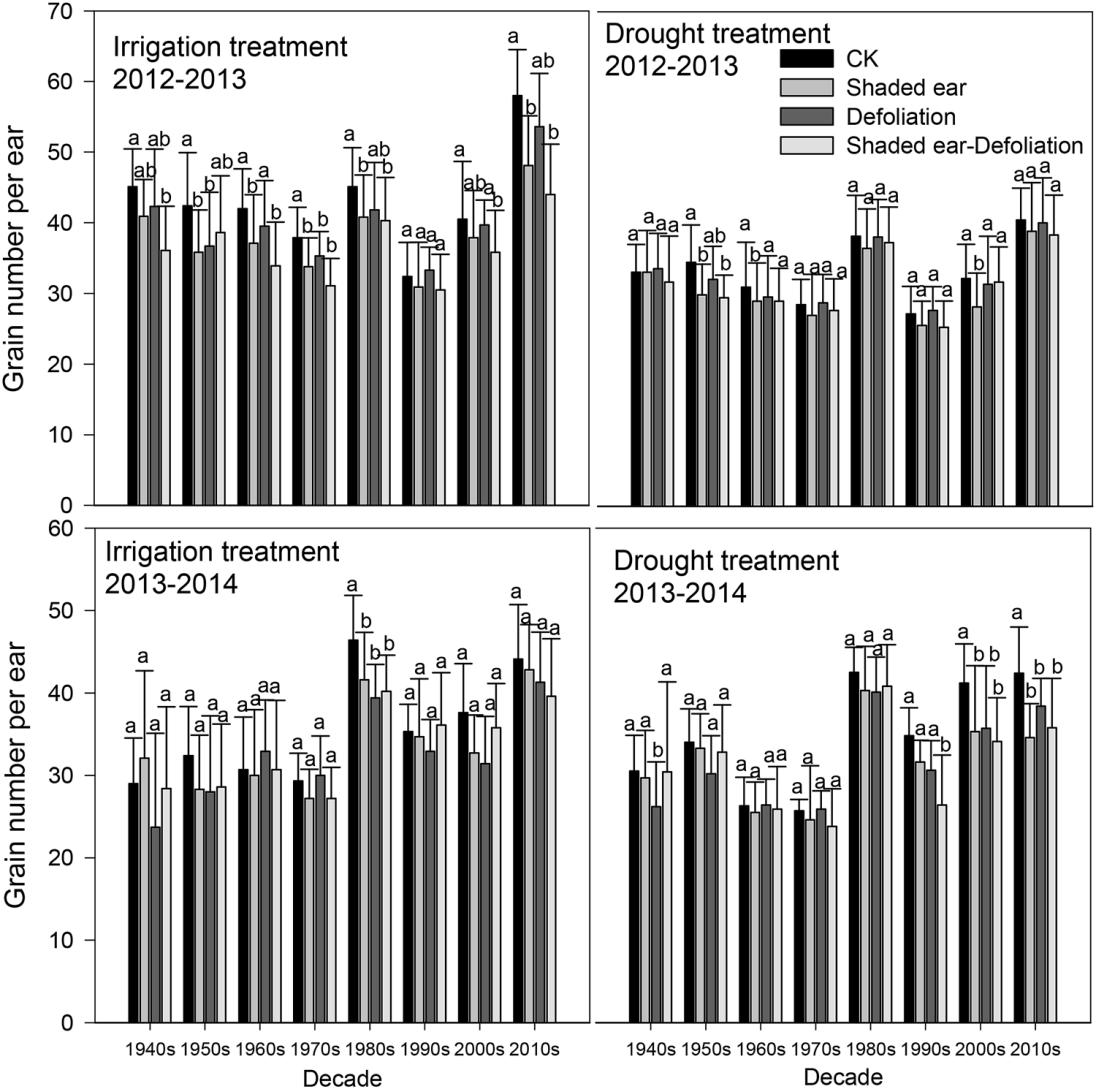
Shaded ear and defoliation influence on grain number per ear of different cultivars. The data represent the mean ± SE (N = 30). The different lowercase letters above the bars denote significant differences for the same cultivar under different defoliation or ear-shading treatments (*P* < 0.05).

Under the two water treatments, the shaded-ear and defoliation treatments all reduced the number of ear grains of all cultivars in both seasons (Fig. 3). During the 2012–2013 season, the ear grains of all cultivars in the CK group and the three treatments were found to be CK > defoliation > shaded-ear > shaded-ear + defoliated. In the 2013–2014 season, most of the cultivars still followed this pattern; only the cultivars of the 1990s and the 2000s under the irrigation treatment showed the order shaded-ear > defoliation. Based on the decrease in the number of ear grains, the amplitude of decrease under the same source-reducing treatment under the irrigation conditions was slightly higher than that under the drought treatment; however, there was no significant difference between the cultivars.

**Figure 4:**
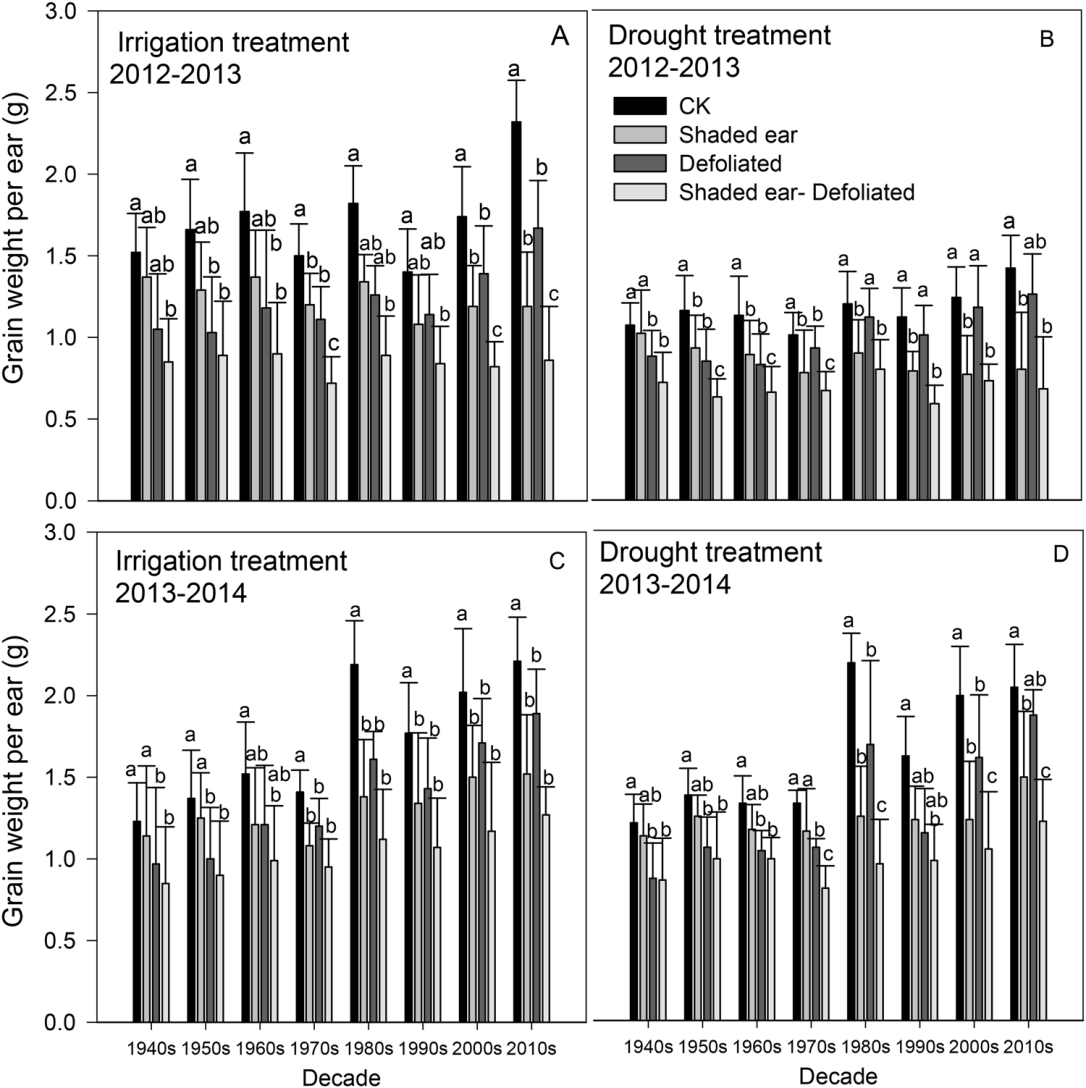
Shaded ear and defoliated influence on grain weight per ear (g) of different cultivars. The data represent the mean ± SE (N = 30). The different lowercase letters above the bars denote significant differences for the same cultivar under different defoliation or ear-shading treatments (*P* < 0.05).

In both seasons, modern cultivars after the 1980s tended to possess a higher grain weight per ear than did the early cultivars; additionally, after experiencing damage to the green organs, such as shaded ear and defoliation, their ear grain weight decreased significantly more than that of the early cultivars (Fig. 4). During the 2012–2013 season, the grain weight per ear of the three modern cultivars cultivated in the 1940s, 1950s and 1960s was as follows: CK > defoliation > shaded-ear > shaded-ear + defoliation. This result showed that the response of grain weight per ear to the damage to the photosynthetic organs was different among different cultivars; the reduction in the ear grain weight of the modern cultivar caused by the shaded ear treatment was larger, and the early cultivars were more reactive to the treatment of leaf cutting. This result is quite different from the result obtained regarding the number of ear grains. In the 2013–2014 season, the difference in grain weight between the different irrigation and drought treatments was not significant.

### Contribution to the grain weight per ear of different green organs after anthesis

**Figure 5:**
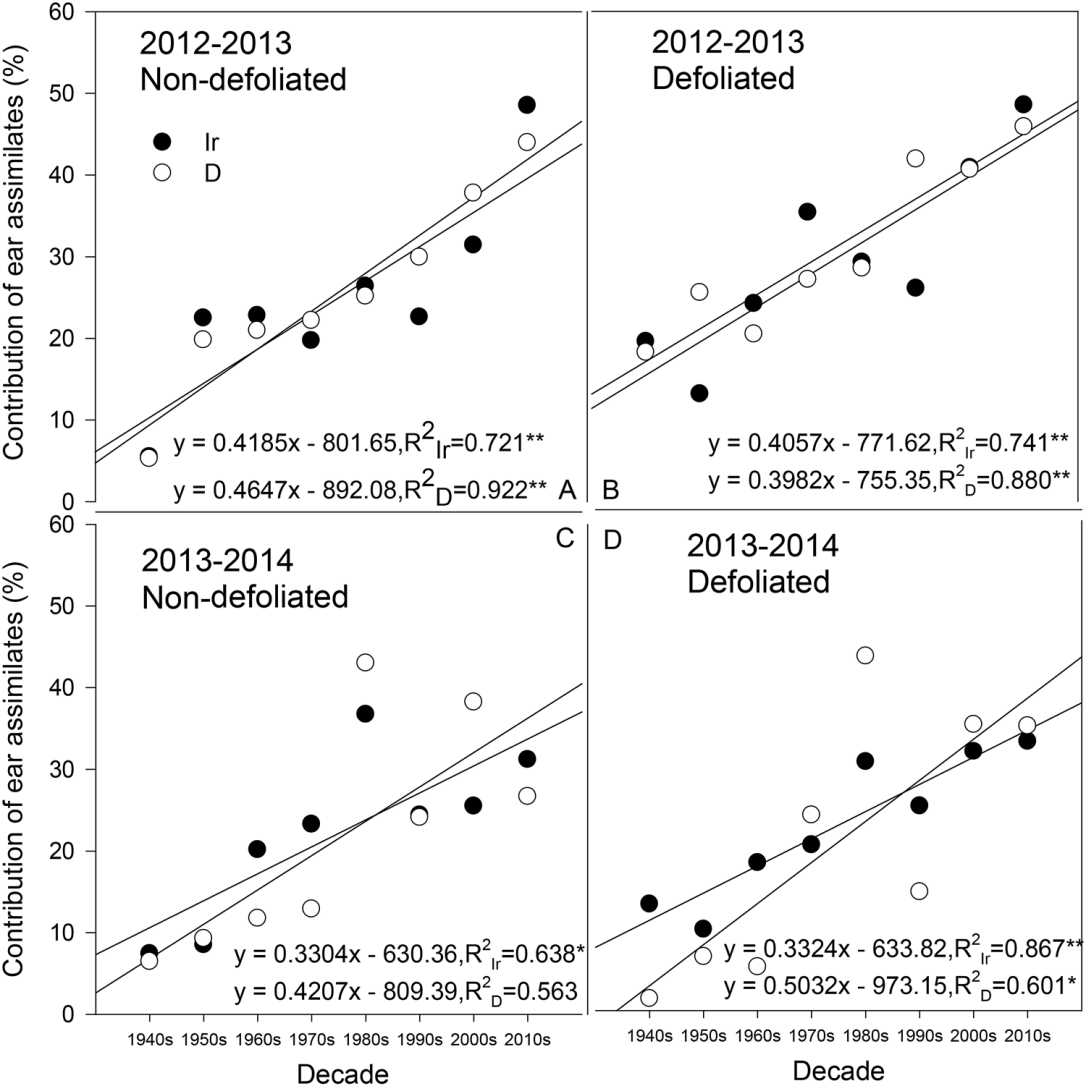
Postanthesis contribution of ear assimilates (CEA) to grain weight in different cultivars. *Significant at *P* < 0.05; **significant at *P* < 0.01; Ir indicates the irrigation treatment; D indicates the drought treatment.

There were significant differences in the contribution of ear to the ear grain weight among the different cultivars (Fig. 5). In the defoliation treatment of 2012–2013, there was a significant linear increase in the CEA under the two water treatments among cultivars from different decades (R^2^_Ir_ = 0.721, R^2^_D_ = 0.922, *P* < 0.01). Among all the cultivars, the Changhan 58 cultivar from the 2010s had the highest CEA of all cultivars, reaching up to 47.15% and 43.96% under the irrigation and drought treatment (Fig. 5A). In this season, most of the cultivars showed a lower CEA under the defoliation treatment than under the nondefoliation treatment, except for the cultivar from the 1950s (Fig. 5B). The trend under the defoliation and nondefoliation treatments was similar, and different cultivars had performed differently in terms of different water treatments. For example, under the nondefoliation treatment, the cultivars in the 1950s, 1960s and 2010s all showed higher CEA in the irrigation treatment than in the drought treatment, while the cultivars in the 1970s, 1990s and 2000s showed the opposite trend. Regarding the other cultivars, the CEA was almost equal under the two water treatments. However, the CEA difference between the different water treatments was relatively small.

During the 2013–2014 season, the CEA of most cultivars decreased compared with the values from the 2012–2013 season (Fig. 5C,D). For example, the highest CEA of Changhan 58 in the 2013–2014 season was just over 30%, which was significantly lower than its value in the previous season by more than 1/4. Although the linear trend slowed, the trend of the increase in the CEA with the cultivar replacement still existed (nondefoliation: R^2^_Ir_ = 0.638, R^2^_D_ = 0.563, *P* < 0.05; defoliation: R^2^_Ir_ = 0.867, *P* < 0.01, R^2^_D_ = 0.601, *P* < 0.05).

**Figure 6:**
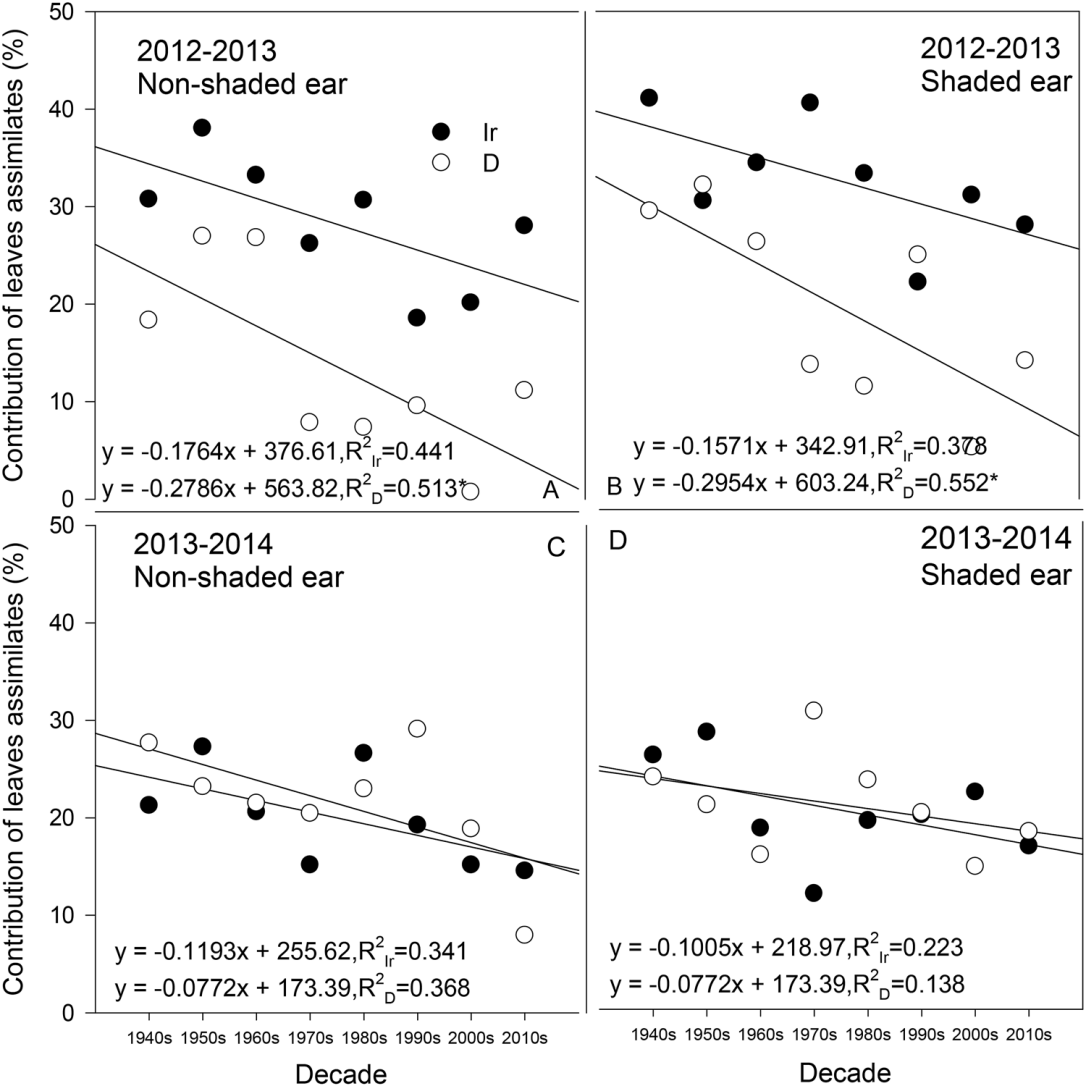
Postanthesis contribution of leaf assimilates (CLA) to grain weight in different cultivars. **Significant at *P* < 0.01; Ir indicates the irrigation treatment; D indicates the drought treatment.

The CLA showed a downward tendency with cultivar replacement and was significantly lower than the CEA (Fig. 6). The most obvious downward trend was shown by the 2012–2013 nonshaded ear treatment (R^2^_Ir_ = 0.441; R^2^_D_ = 0.513, *P* < 0.05; Fig. 6A). The CLA of all cultivars was obviously higher under irrigation than under drought. The general trend of the CLA under the shaded-ear treatment of 2012–2013 was consistent with that under the nonshaded ear treatment, all of which showed a downward trend with the cultivation decades, especially under the drought treatment (R^2^_D_ = 0.552, *P* < 0.05). In comparison, the CLA under the shaded-ear treatment increased relative to that under the nonshaded ear treatment, especially for Mazha in the 1940s, which showed an increased CLA under the shaded-ear treatment of more than 10% under both water treatments.

In the 2013–2014 season, most of the cultivars had a CLA of approximately 30% (Fig. 6C, D), while the decreasing trend of CLA over time was still significant.

### The contribution of preanthesis stem assimilates

**Figure 7:**
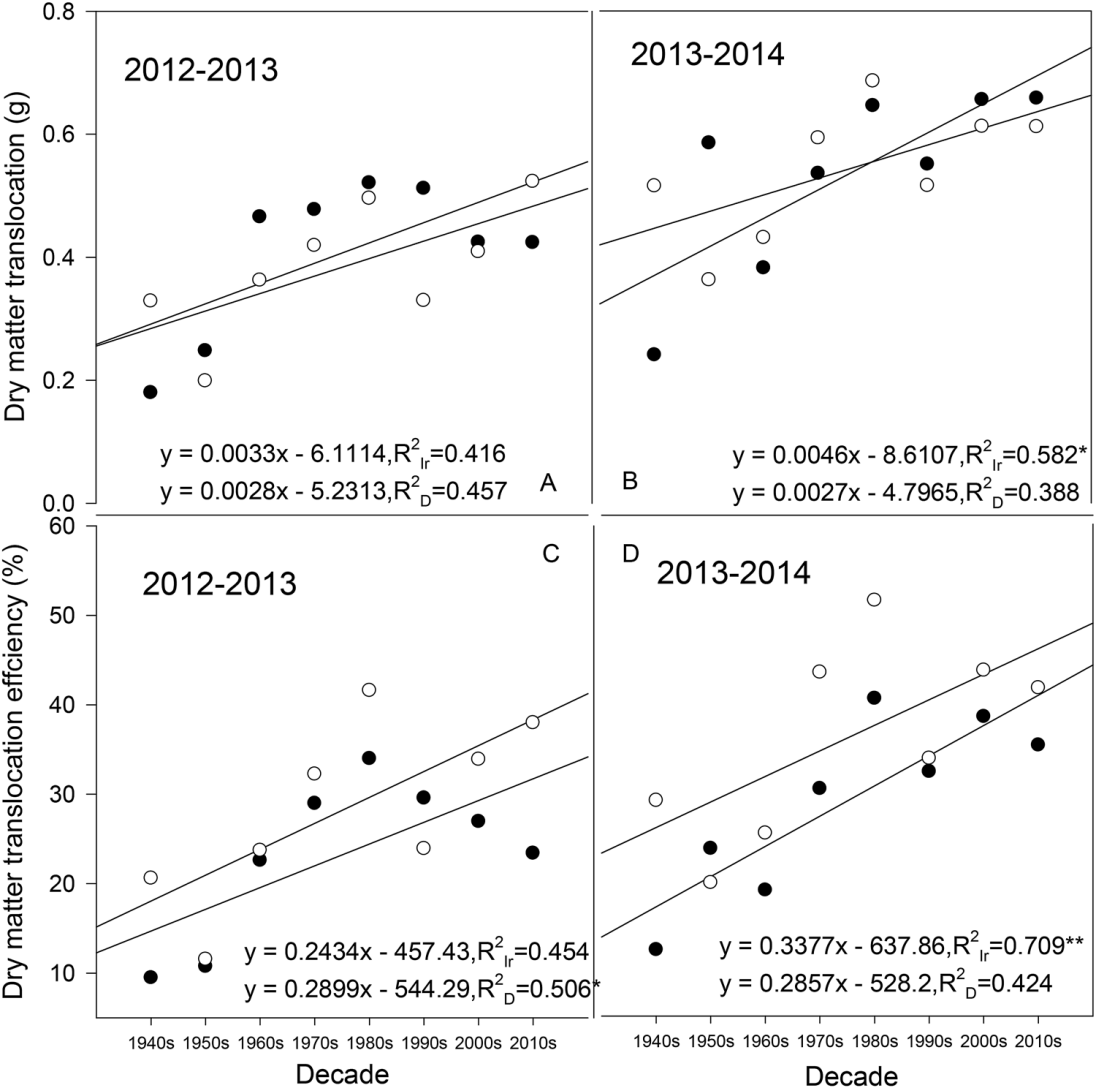
Dry matter translocation (DMT) and dry matter translocation efficiency (DMTe) in different cultivars. *Significant at *P* < 0.05; **significant at *P* < 0.01; Ir indicates the irrigation treatment; D indicates the drought treatment.

Within the two planting seasons, 2012–2013 and 2013–2014, DMT and DMTe showed an increasing trend with cultivation decade (Fig. 7); moreover, DMT showed the most evident increasing trend under the irrigation treatment in 2013–2014 (R^2^_Ir_ = 0.582, *P* < 0.05), which was similar to that of DMTe (R^2^_D_ = 0.709, *P* < 0.01). The DMT and DMTe differences between the drought and irrigation treatments were not consistent; DMT was always higher under the irrigation treatment in the 2012–2013 season, while in 2013–2014, half of the cultivars showed a higher value under drought than under irrigation (Fig. 7C,D).

**Figure 8:**
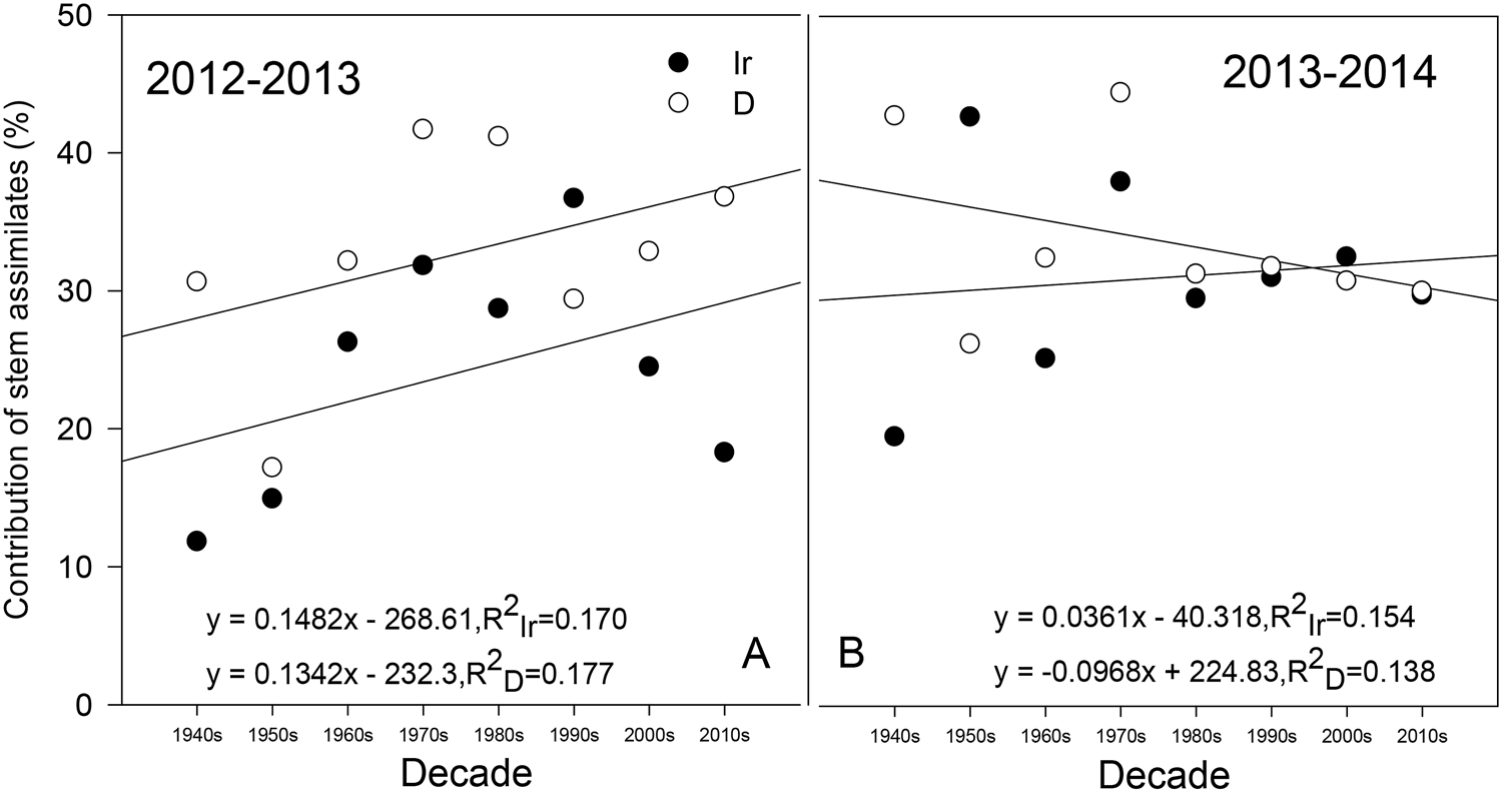
Contribution of preanthesis stem assimilates (CSA) to grain weight in different cultivars. *Significant at *P* < 0.05; ** significant at *P* < 0.01; Ir indicates the irrigation treatment; D indicates the drought treatment.

The CSA showed different trends between 2012–2013 and 2013–2014 (Fig. 8). In the 2012–2013 season, all cultivars had a high CSA under the drought treatment except for Jinmai 33 in the 1990s (Fig. 8A). Although the CSA of the modern cultivars tended to be higher than that of the early cultivars under both water treatments, the trend of the increasing CSA with the cultivation decade was not significant (R^2^_Ir_ = 0.170, R^2^_D_ = 0.177, *P* > 0.05). In the 2013–2014 season, except for the cultivars from the 1950s and 2000s, all cultivars showed higher CSA values under drought than under irrigation. The CSA still tended to increase slightly with cultivar replacement under the irrigation treatment (R^2^_Ir_ = 0.154, *P* > 0.05), while under the drought treatment, a contrasting trend was observed (R^2^_D_ = 0.138, *P* > 0.05).

### Relationship among the CLA, CEA and biological characteristics

It can be concluded that there was no stable or obvious linear relationship between grain weight per spike and CLA (Table 5); however, there was a clear positive correlation between grain weight per spike and CEA, with the correlation coefficient between ear grain weight and CEA reaching up to 0.889 (*P* < 0.01) in the 2013–2014 irrigation treatment. Finally, there was a negative correlation between CEA and CLA, which means that a cultivar with a high CEA usually has a low CLA. DM_max_ and DMT_e_ showed a negative correlation with CLA but a positive correlation with CEA and grain weight per spike, especially from 2013 to 2014. No stable correlation between CSA and grain weight per spike, CLA or CEA was observed.

**Table 5:** Correlation of the ear and leaf contribution to grain weight with biological indicators.

Growing season	CLA	CEA	DMT	DMTe	CSA
**2012–2013**					
Ir					
Grain weight per spike	0.204	0.841**	0.123	0.081	− 0.331
CLA		− 0.19	− 0.503	− 0.589	− 0.611
CEA			0.448	0.399	0.08
D					
Grain weight per spike	− 0.186	0.779*	0.505	0.502	0.059
CLA		− 0.523	− 0.668	− 0.779*	− 0.679
CEA			0.555	0.796*	0.251
**2013–2014**					
Ir					
Grain weight per spike	− 0.21	0.889**	0.759*	0.804*	− 0.015
CLA		− 0.269	− 0.123	− 0.407	0.091
CEA			0.676	0.912**	0.004
D					
Grain weight per spike	− 0.488	0.950**	0.732*	0.750*	− 0.505
CLA		− 0.27	− 0.341	− 0.347	0.247
CEA			0.763*	0.797*	− 0.406

With the same increasing or decreasing trend, data that did not include the shaded ear in the leaf contribution to grain weight were solely considered and data that did not include defoliation in the ear contribution to grain weight were solely considered; data regarding the CK treatment for grain weight per spike were only considered; *significant at *P* < 0.05; **significant at *P* < 0.01.

## Discussion

Source, sink and flow are three key factors that affect the yield formation of wheat, and the wheat yield is increased with an abundant source, large sink, and smooth flow^28–30^. In our study, shaded ear and defoliation after anthesis both reduced the grain weight and number of grains per ear of all cultivars (Figs. 3, 4, *P* < 0.05). These results indicate that both leaf and ear are important photosynthetic sources after anthesis for wheat, and the function damage of leaf and ear may reduce the size of the wheat sink. Most cultivars showed a greater decline from shaded ear than from defoliation, meaning the ear may be more important as a photosynthetic source than the leaf after anthesis.

Modern cultivars are always large-spike cultivars with high grain numbers, which may result in source limitations when the leaves are damaged^24^; these conditions could make the nonleaf photosynthetic organs, such as the ear and stem, important for photosynthetic accumulation. There are significant differences in CEA between different cultivars; specifically, cultivars cultivated after the 1980s showed a greater decrease under shaded-ear treatment than the earlier cultivars (Fig. 3), which could indicate that the contribution of the ear to photosynthetic accumulation after anthesis is more important for modern cultivars than for earlier cultivars. CEA showed a significant increase with cultivar replacement, and its peak value reached approximately 50% (Fig. 5); furthermore, CEA showed a significant positive correlation with grain weight under both irrigation and drought treatments (Table 3). The results corroborated the hypothesis that dryland wheat breeding has enhanced the proportion of ear photosynthesis in modern cultivars in Shaanxi Province. This conclusion was consistent with the results of Maydup et al. (2012) in Argentina^15^ but differed from the results of Sanchez-Bragado et al. (2014) in Spain^10^. Most modern wheat in Shaanxi Province of China are middle-spike or large-spike cultivars, characterized by an increased ear photosynthetic area, which may be one of the reasons for the increase in CEA in the modern cultivars. The stem weight of cultivars without dwarf genes (i.e., 1940s, 1950s, 1960s, 1990s) continued to increase after anthesis, and some of the photosynthetic products were consumed, which may reduce the transport efficiency of assimilates to grains. Furthermore, CEA and CLA showed a slight negative relationship in both experimental seasons under both irrigation and drought treatments, indicating that the key photosynthetic source changed from leaf preanthesis to ear after anthesis. As the ear is the photosynthetic organ that has the most delayed senescence in wheat^15^, the relative change between the different photosynthetic sources may be more beneficial to the dry weight accumulation of grains in the late filling stage of modern cultivars.

Wheat can accumulate considerable amounts of carbohydrates and nitrogenous compounds in the stem before anthesis^19,31^, and the plant reallocates the accumulated carbon and nutrients among different tissues during the grain-filling period^32^. The temporary storage of accumulations in the stem is especially important when the function of other photosynthetic organs is limited^33^. Because the reactivation transport rate is far greater than the respiratory rate of consumption^34^, DMT could be used to evaluate the transport volume of water-soluble carbohydrates during grain-filling^35^. DMTe can account for 10–50% of the yield components based on the different genotypes and environmental conditions^19,23^. In our study, even with the photosynthetic limitation of both leaf and ear, wheat can still maintain a certain grain yield per ear (Fig. 4). In the experimental season of 2013–2014, there was considerable precipitation after anthesis, and the DMTe and CSA were slightly higher than the values in 2012–2013, which enhanced the importance of the stem (sheath included) for yield accumulation. During the replacement of cultivars, although the plant height and the maximum dry weight of a single stem after anthesis significantly decreased (Fig. 2), DMT and DMTe both increased significantly (Fig. 7). However, with the improved grain weight of modern cultivars, no significant difference in CSA was found between the selected cultivars (Fig. 8). Even with the dwarf height and the decreased stem weight, modern cultivars possess higher DMT and DMTe, and the distribution of photosynthetic products after anthesis is more balanced, which can be beneficial in terms of maintaining the yield despite the limitations of the ear and leaf.

Water deficits can influence the wheat dry matter distribution of different organs and affect the transport of carbohydrates to grains^36^. Drought could improve the ear contribution to grain weight, which would indicate that the ear may be more accommodative to water deficit conditions. Drought promoted the DMTe of dryland wheat in Shaanxi Province of China, as well as the CSA (Figs. 7, 8), while the CLA always decreased under the drought treatment (Fig. 6). However, in the same experimental season, the CEA was almost unaffected (Fig. 5). The results showed that stem dry matter translocation was significantly affected by water deficits and that the leaf was more sensitive to water conditions than the ear as a photosynthetic organ.

Although the influence of water treatment on the contribution of different green organs to grain weight was not consistent between different experimental seasons, the contribution of leaves was more influenced by water than that of the ear and stem. Additionally, CLA may even show more significant reductions under drought conditions compared with irrigation treatment (Fig. 6), indicating that yield may be easier to maintained with the selection of cultivars with higher ear and stem contributions under the condition of water deficit. Manipulative treatments, such as defoliation and shading of photosynthetic organs, may trigger unwanted compensatory mechanisms^36^. Compared with CK, under the combination of defoliation and shaded ears, the grain weight and grain number per ear decreased less than the total decrease that was caused by the separate defoliation and shaded-ear treatments, indicating that compensatory mechanisms of another type were involved (Figs. 3, 4). Although compensatory capacity may differ among different genotypes, we have not accurately determined the difference or provided a quantitative evaluation; therefore, the contribution of different organs to grain weight in our study may be underestimated.

## Conclusion

Once the dwarf genes were introduced, the plant height and stem dry weight of dryland wheat in Shaanxi Province decreased significantly during the cultivar replacement process; however, there was also a remarkable increase in DMT and DMTe. The risk of lodging was reduced, but the contribution of the assimilates in the stem to yield was increased. Leaf, ear and stem are all important dry matter accumulations of yield for dryland wheat, but modern cultivars tended to possess higher CEA, DMT and DMTe, along with lower CLA. The ear was more influential as a sink, and CEA was less sensitive to the water conditions, indicating a greater importance of ear assimilative capacity in the future study of “source” constraints. In the future breeding process in Shaanxi Province, cultivars with a stronger capacity for green organs, especially greater CEA, are expected to increase the grain yield.
